# Notes and News

**Published:** 1896-07-18

**Authors:** 


### Notes and News.

(Collected and Communicated.)

Guy's Hospital has received a donation of £200 from Lord Salisbury and of £300 anonymously during the last week.

Three prizes of £50, £30, and £20 respectively are offered by the Joint Hospital Committee for plans for the new isolation hospital for Richmond, Heston, and Isleworth Urban District.

There is a proposal on foot to perpetuate the memory of the poet Keats by endowing a bed at Guy's Hospital to bear his name. Keats was for a short time a medical student at Guy's, and it is thought that the memorial would be most suitable. It would also assist to lighten the burden placed upon the resources of the hospital.

At the recent quarterly meeting of the Council of the Royal College of Surgeons, Mr. T. Bryant, Mr. J. Davies Colley, and Mr. T. Pickering Pick were elected members of the council. A report from the Committee on the dental surgery regulations was received and adopted. Under this new scheme there will be three examinations—a preliminary science examination and two professional examinations for the licence.

The latest decision of the board of managers of the Chesterfield Hospital is that, seeing that the institution was established for surgical cases, and that the beds are in demand for such, they cannot entertain the proposal that they should admit medical cases. It is very evident, however, that hospital accommodation for medical cases is badly wanted at Chesterfield, and the inhabitants will have to enforce this view upon the committee, unless they are prepared to face the expense of building a new hospital, and all the evils of a duplicate staff.

We are glad to note that our article on Street Collections has already borne practical fruit. In reply to Mr. Hogan, the Home Secretary has promised that these street collections shall in the future be carefully watched by the police. It is also a matter for congratulation that several members of Parliament testified, in the House of Commons on Monday, to the annoyance caused by street collections, and to the ease with which they may be used for fraudulent purposes. We hope the Council of the Hospital Saturday Fund will promptly facilitate their abolition by resolving to discontinue street collections for the future.

The Birmingham Hospital Saturday collection has exceeded the most sanguine expectations, and has already reached upwards of £15,000.

Dr. Samuel Wilks, President of the Royal College of Physicians, has been appointed Physician Extraordinary to Her Majesty the Queen, in place of the late Sir George Johnson.

The Metropolitan Hospital Sunday Fund collection amounts now to £42,000. From the Jewish community nearly £1,000 has been received. An anonymous donor, whose initials are J. C., sends £200.

The Convalescent Home for Children, presented to the Birmingham Hospital Saturday Fund by an anonymous donor, has been formally opened. The guests who journeyed to Bryn Marle were entertained at lunch by Mr. and Mrs. Smedley. For the present year the cost will not fall upon the fund, but after that time it is expected that the committee will take it over.

The Convalescent Dinners' Society, of which Lord Chelmsford is chairman, appeals for further contributions. The object of the society is to provide dinners for patients dismissed as convalescent from the hospitals, who are much in need of good food to assist in the complete re-establishment of their health. Fourteen daily dinners have been distributed to 1,000 poor convalescents. Such a work, judiciously managed in conjunction with the hospital authorities, could not fail to do a most useful work.

The death of Major Roderick Owen, of the Nile Expedition, from cholera, has cast a gloom over the camp, as he was reported to have been in excellent health previous to the attack. He himself was most actively engaged in superintending preventive measures, and had spared no pains to render these as complete as possible. The last cholera returns are regarded as very satisfactory. A number of military medical officers have been ordered out to reinforce the existing staff, and some twenty non-commissioned officers of the Medical Staff Corps will also proceed to Egypt.

We are glad to notice that Mr. Chamberlain, in his speech on municipal government, dwelt especially upon the importance of the adequate remuneration of the permanent officials who preside over the various departments of a municipality. He well said: "There is no economy more disastrous than the economy which endeavours to make cheeseparing savings in the remuneration of men whose services may be priceless." We wish we could impress this more deeply upon the minds of those who are immediately responsible for the management of the great hospitals and charities in this country. What is true of the heads of departments in municipal life is equally true of the higher officials to whose devotion we owe the efficiency of our hospitals and larger charities. Such institutions, like the municipality, cannot afford to have second-rate officials, and as the representatives of progress and the highest administrative efficiency we would impress upon the managers that if they want to have first-rate officials, as Mr. Chamberlain truly said, they must pay the price that competition has established for the particular kind of service which it is all-essential they should obtain in the management of the institution for which they are responsible.

